# A systematic review and thematic synthesis of Canada’s LGBTQ2S+ employment, labour market and earnings literature

**DOI:** 10.1371/journal.pone.0223372

**Published:** 2019-10-02

**Authors:** Sean Waite, John Ecker, Lori E. Ross

**Affiliations:** 1 Department of Sociology, the University of Western Ontario, London, Ontario, Canada; 2 Canadian Observatory on Homelessness, Toronto, Ontario, Canada; 3 Dalla Lana School of Public Health, University of Toronto, Toronto, Ontario, Canada; Università degli Studi di Perugia, ITALY

## Abstract

**Background:**

The last two decades have witnessed a considerable growth in the literature focusing on LGBTQ2S+ employment, labour market inequality, and income. During the same period, Canada has emerged as a trailblazer in employment protections for both sexual and gender minorities. Unfortunately, the Canadian literature on LGBTQ2S+ employment outcomes and experiences is disperse and underdeveloped.

**Objective:**

This paper brings together this disperse research and provides the first systematic review of Canada’s LGBTQ2S+ employment and earnings literature.

**Methods:**

We start with a systematic review and thematic synthesis of the broadly defined literature on LGBTQ2S+ poverty in Canada. We use a thematic synthesis to isolate the LGBTQ2S+ literature on employment, labour market inequality, and earnings. Our search of electronic databases took place in April 2018 and was updated in January 2019.

**Results:**

A total of 532 abstracts and full texts were screened by reviewers, which resulted in 84 articles included in our final sample. These articles were then sorted by keywords and those pertaining to employment, labour market inequality, and income (n = 31) were included in this analysis. While estimates of sexual minority wage gaps vary depending on the data and methods used, most studies have found wage penalties for gay men and wage premiums for lesbians, relative to their heterosexual counterparts. The literature on bisexual employment is particularly scant but finds that bisexual men and women also earn less than their heterosexual counterparts. Research on the subjective workplace experiences of LGBTQ2S+ individuals find unique challenges, barriers and, at times, exclusion from the Canadian labour market.

**Conclusions and implications:**

While the literature on LGBTQ2S+ employment outcomes and experiences in Canada is growing, much is left unknown. The principal limitation for researchers continues to be the dearth of population-based surveys that include questions on sexual orientation, gender identity, and relevant employment characteristics. To date, few studies have explored employment outcomes or the subjective workplace experiences of bisexuals, transgender, two-spirit or other gender minority peoples.

## Introduction

There is a growing international literature interested in the lives of lesbian, gay, bisexual, transgender, queer, and two-spirit (a term that captures Indigenous individuals who identify as gay or lesbian, transgender, or occupy multiple gender categories and sexualities) [1] (LGBTQ2S+) individuals. An area receiving increasing attention is employment, labour market inequality, and earnings. Work can be central to an individual’s well-being, providing not only a source of income but also structure, meaning, and identity. Income from gainful employment also provides food, clothing and safe and secure housing.

To date, most of the LGBTQ2S+ employment literature has come from the United States. While this literature has made valuable contributions and advanced our knowledge on the employment outcomes and experiences of LGBTQ2S+ individuals, other countries may provide unique insights and interesting cases for analysis. One such country is Canada. Canada has been a trailblazer in providing employment protections on the basis of sexual orientation [2]. In 1977, Quebec became the first province in Canada to include sexual orientation in their Provincial Charter of Human Rights and Freedoms, which protects against employment and housing discrimination. Over the next few decades, other provinces made similar amendments. At the Federal level, the Canadian Human Rights Act was amended to include sexual orientation in 1996. During 2010s’ these acts were further amended to include gender identity and by 2017, all provinces included gender identity as a protected status in employment and housing. During this period, attitudes toward sexual minorities also improved [3,4] and by international comparison, Canada ranks low on the World Values Survey’s measures of homonegativity [5]. Canada was also the first country outside of Europe to legalize same-sex marriage in July 2005.

The Canadian case is arguably one of strong legal protections and relatively tolerant attitudes [2–5]. Unfortunately, much of the Canadian literature on LGBTQ2S+ employment has been dispersed across several fields, such as political science, medicine, economics, sociology and geography. To date there has been no attempt to synthesize these findings. Studies on gay, lesbian and bisexual earnings have also produced varying estimates of sexual minority wage gaps, depending on the data and methods employed. We attempt to reconcile theses inconsistencies in this paper.

This paper presents the first systematic review of the LGBTQ2S+ employment literature in Canada. In particular, we are guided by three main research questions. First, is sexual orientation an important dimension of labour market stratification in Canada? Second, what are the employment outcomes and experiences of Canada’s LGBTQ2S+ community? And lastly, what are the current limitations and opportunities for future research in this field? To answer these questions, we conduct a systematic review of the literature related to LGBTQ2S+ poverty, broadly defined. We then conduct a thematic synthesis and focus our results on research pertaining to LGBTQ2S+ employment, labour market inequality, and income. Lastly, we reflect on data limitations and research opportunities for advancing the LGBTQ2S+ employment literature into the future.

## Methods

This systematic review and thematic synthesis of the LGBTQ2S+ employment literature is drawn from a larger systematic review of the Canadian LGBTQ2S+ poverty literature, broadly defined. Poverty is multifaceted and includes economic, social, and political dimensions [6–8]. Poverty has many consequences, including poor health, mortality, lack of education, inadequate housing, homelessness, increased risk of violence, and discrimination [6–8]. Our systematic review starts with a broad definition of poverty and then conducts a thematic synthesis to focus on the Canadian literature pertaining to LGBTQ2S+ employment, labour market inequality, and earnings.

### Search strategy

We conducted a systematic search for literature related to LGBTQ2S+ poverty in Canada. The search was limited to articles written in either English, French or Spanish. We are not aware of any literature omitted because of this language specification. The initial search took place in April 2018. First, we conducted database searches using Medline, PsychINFO, Sociological Abstracts, and EconLit with subject headings, titles, or abstract terms for LGBTQ2S+ (“sexual minority”, homosexual*, transgender*, queer* etc.), poverty (poverty “homeless people”, poor, welfare, education*, “wage gap” etc.), and Canada (Canada, Canad*, Ontario, Toronto, Montreal, Vancouver). For each database search, we used roughly 30 LGBTQ2S+ related terms, 25 poverty related terms and 20 Canada related terms (See S1 Appendix for a list of search keywords). For example, the following search strategy was used for the Sociological Abstracts database: ((su(sexual orientation) OR su(sexual identity) OR su(sex role identity) OR su(bisexual*) OR su(lesbian*) OR su(homosexual*) OR su(sexual preference*) OR su(sexual behaviour) OR su(sexual behavior) OR su(gays lesbians) OR su(transgender persons) OR su(transsexual*) OR su(gender identity) OR su(sexual minority) OR su(transsexual*)) OR (ab(sexual minority) OR ab(lesbian*) OR ab(gay) OR ab(bisexual*) OR ab(non-monosexual) OR ab(monosexual) OR ab(plurisexual*) OR ab(men who have sex with men) OR ab(MSM*) OR ab(women who have sex with women) OR ab(WSW*) OR ab(transgender*) OR ab(transsexual*) OR ab(queer) OR ab(two-spirit*) OR ab(LGB*) OR ab(BLB*) OR ab(sexual orientation) OR ab(gender identity)) OR (ti(sexual minority) OR ti(lesbian*) OR ti(gay) OR ti(bisexual*) OR ti(non-monosexual) OR ti(monosexual) OR ti(plurisexual*) OR ti(men who have sex with men) OR ab(MSM*) OR ti(women who have sex with women) OR ti(WSW*) OR ti(transgender*) OR ti(transsexual*) OR ti(queer) OR ti(two-spirit*) OR ti(LGB*) OR ti(BLB*) OR ti(sexual orientation) OR ti(gender identity))) AND ((su(poverty) OR su(homeless people) OR su(homelessness) OR su(low income groups) OR su(low income) OR su(poor) OR su(wage gap*) OR su(education*) OR su(employ) OR su(unemployed) OR su(welfare) OR su(shelters) OR su(income) OR su(financial support) OR su(wealth) OR su(housing) OR su(antipoverty programs) OR su(deprivation) OR su(social class) OR su(socioeconomic status) OR su(occupation)) OR (ab(poverty) OR ab(poor) OR ab(wage gap*) OR ab(income) OR ab(unecmploy*) OR ab(employ*) OR ab(homeless*)) OR (ti(poverty) OR ti(poor) OR ti(wage gap*) OR ti(income) OR ti(unecmploy*) OR ti(employ*) OR ti(homeless*))) AND ((su(Canad*) OR su(Toronto) OR su(Montreal) OR su(Ontario) OR su(Quebec) OR su(Vancouver) OR su(British Columbia) OR su(Alberta) OR su(Manitoba) OR su(Saskatchewan) OR su(New Brunswick) OR su(Nova Scotia) OR su(Prince Edward Island) OR su(Newfoundland*) OR su(Yukon) OR su(Northwest Territories) OR su(Nunavut)) OR (ab(Canad*) OR ab(Toronto) OR ab(Vancouver) OR ab(Montreal)) OR (ti(Canad*) OR ti(Toronto) OR ti(Vancouver) OR ti(Montreal))). These searches resulted in 438 articles from database searches. Four studies did not get captured by the database searches and were included by hand, bringing the total to 442 articles for screening.

To ensure the most up-to-date literature review, we conducted a follow-up search in January 2019 to capture publications between April 2018 and the end of December 2018. Replicating the same search methodology as above, we found an additional 88 publications through the same database searches. Two additional articles were included that were not captured by the previous database searches. The follow-up literature review produced 90 additional references for screening.

### Inclusion criteria and quality assessment

To ensure an exhaustive review of the LGBTQ2S+ poverty literature, we used very broad inclusion criteria. First, studies had to include some form of data, such as qualitative, quantitative, or secondary data. Second, studies had to report data on sexual or/gender minority people, which we defined broadly. For example, our review included roughly 30 LGBTQ2S+ terms. Third, the study must have reported on poverty or relative indicators, which we again defined broadly using 25 poverty related terms. Fourth, studies must have used either primary or secondary Canadian data. Fifth, the study had to be published in either English, French or Spanish. Lastly, there were no restrictions on publication date, type, or source, i.e. peer-reviewed vs. non-peer-reviewed.

During the screening phase, some additional inclusion/exclusion criteria were made. We excluded historical analysis since these studies were unlikely to provide relevant information on current LGBTQ2S+ poverty experiences. We did not conflate LGBTQ2S+ sex work with poverty and only included studies on sex workers that addressed one of our poverty measures. We included studies composed of HIV positive participants only when it was possible to isolate LGBTQ2S+ respondents and data on poverty related measures were also gathered. Similarly, studies on LGBTQ2S+ health outcomes were included when this was linked to poverty or related variables. Studies reporting data on multiple countries, including Canada were included if the Canadian data/findings could be isolated.

During the first phase of screening in May 2018, we reviewed articles and removed duplicates (n = 73) and irrelevant studies (n = 263). The remaining 106 studies underwent a full-text review to assess their eligibility. To be included in the study, each text had to be reviewed by at least two researchers and deemed relevant. If both reviewers deemed the study irrelevant, it was excluded. If there was disagreement as to whether the study should be included, a third researcher cast the deciding vote.

We use the PRISMA guidelines as a framework for this analysis [9]. Overall, there were few conflicts over which studies should be included in the final sample. During the full-text screening, 37 additional studies were excluded. These studies were excluded for the following reasons: historical study (n = 16), no LGBTQ2S+ data reported (n = 10), no Canadian data reported (n = 6), no poverty-related data reported (n = 3), no original data/analysis (n = 1) and wrong outcome (n = 1). This resulted in 69 articles related to LGBTQ2S+ poverty in Canada.

**Fig 1:**
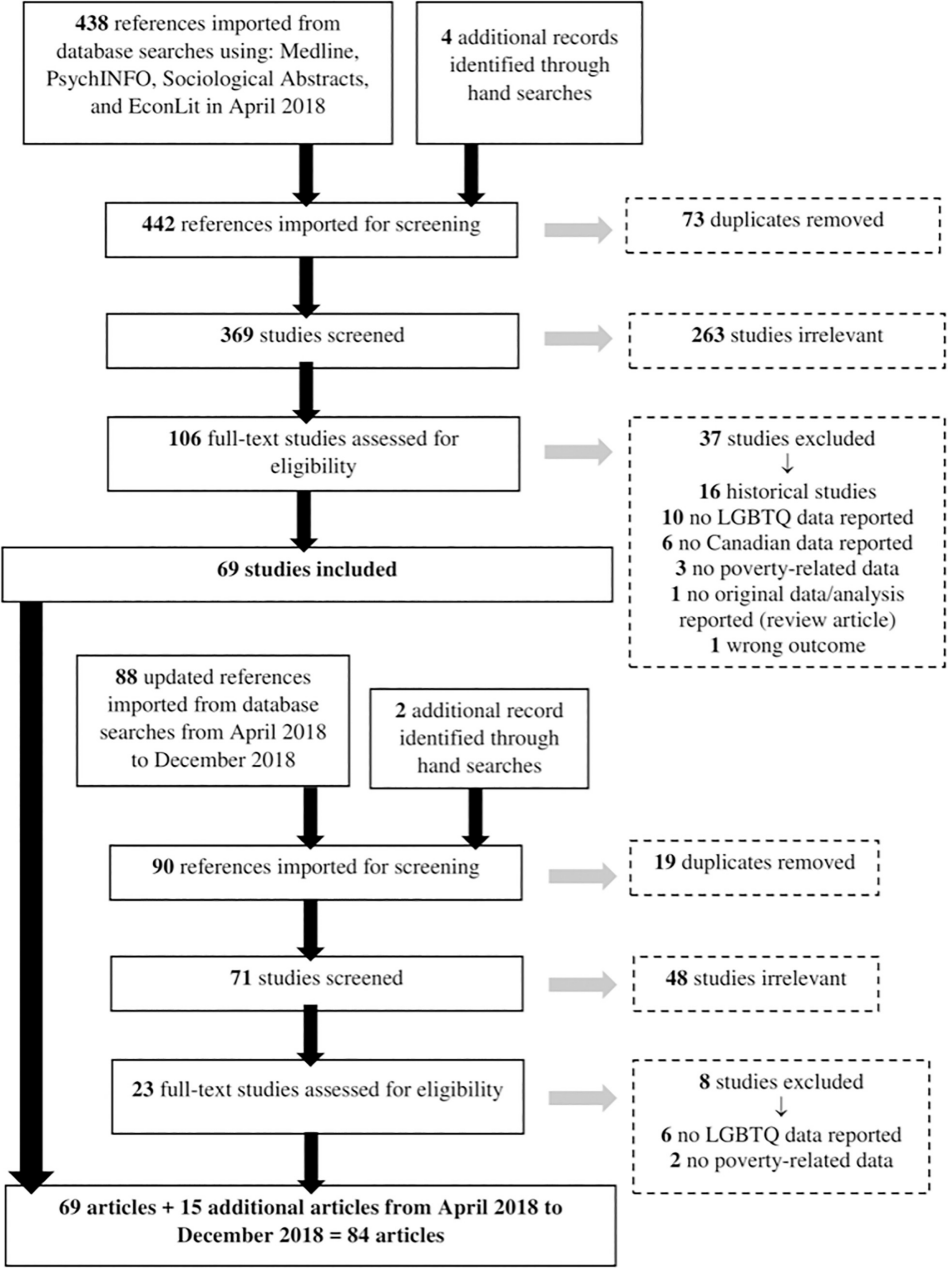
Search strategy flowchart.

The follow-up search followed the same inclusion criteria as above but was restricted to the period from April 2018 to the end of December 2018. Again, we removed duplicates (n = 19) and irrelevant studies (n = 48). After these exclusions, 23 studies remained for full-text review. These articles were reviewed by a single reviewer who removed studies that did not have LGBTQ2S+ (n = 6) or poverty (n = 2) related data. This resulted in 15 additional articles to be included in the review. In total, there were 84 articles related to LGBTQ2S+ poverty included in the study. Fig 1 provides a visual representation of the systematic review methodology.

### Data extraction

We assigned keywords to the final 84 articles based on their particular research focus. For example, health outcomes; life course; education; housing/homelessness; and, employment, income, and the labour market. In most cases, articles received multiple keywords, such as life course and education or health outcomes and housing/homelessness. We filtered the extracted texts and isolated only those that included keywords employment/income/labour markets (n = 31). Table 1 includes a list of the articles included in our systematic and thematic review.

**Table 1:** Articles included in the systematic review and thematic synthesis.

Author(s):	Title:	Journal / Publication:
Adam (1981) [10]	Stigma and Employability: Discrimination by Sex and Sexual Orientation in the Ontario Legal Profession	The Canadian Review of Sociology and Anthropology
Adam and Rangel (2015) [11]	The post-migration sexual citizenship of Latino gay men in Canada	Citizenship Studies
Allen (2015) [12]	Household Production and Sexual Orientation	Economic Inquiry
Bauer and Scheim (2015) [13]	Transgender People in Ontario, Canada: Statistics from the Trans PULSE Project to Inform Human Rights Policy	Report
Bowring and Brewis (2009) [14]	Truth and consequences: Managing lesbian and gay identity in the Canadian workplace	Equal Opportunities International
Brennan et.al. (2013) [15]	Socio-demographic profile of older adults with HIV/AIDS: gender and sexual orientation differences.	Canadian Journal on Aging
Brown (1998) [16]	Sexual Orientation and Labor Economics	Feminist Economics
Card et.al. (2018) [17]	A latent class analysis of substance use and culture among gay, bisexual and other men who have sex with men.	Culture, health & sexuality
Carpenter (2008) [18]	Sexual Orientation, Work, and Income in Canada	Canadian Journal of Economics
Cerf (2016) [19]	Sexual orientation, income, and stress at work.	Industrial Relations
Cotter (2016) [20]	Sexual Misconduct in the Canadian Armed Forces, 2016	Statistics Canada
Couto (2014) [21]	Covered in Blue: Police Culture and LGBT Police Officers in the Province of Ontario	MA Thesis, Royal Roads University
Denier and Waite (2017) [22]	Sexual Orientation Wage Gaps across Local Labour Market Contexts: Evidence from Canada	Industrial Relations
Denier and Waite (2016) [23]	Data and Discrimination: A research note on sexual orientation in the Canadian labour market	Canadian Studies in Population
Dilmaghani (2018) [24]	Sexual Orientation, Labour Earnings, and Household Income in Canada	Journal of Labour Research
Dilmaghani (2018) [25]	Sexual orientation, labour supply and occupational sorting in Canada	Industrial Relations Journal
Ferlatte et.al. (2018) [26]	An Application of Syndemic Theory to Identify Drivers of the Syphilis Epidemic Among Gay, Bisexual, and Other Men Who Have Sex With Men.	Sexually transmitted diseases
Fournier (2005) [27]	Homosexuality in the Army and Police: Progress Achieved and Experiments Lived by Gay Soldiers, Police Officers, and Gay Police According to their Own Point of View (English translation)	Dissertation Abstracts International
Harris (2013) [28]	Essays in Applied Econometrics	Dissertation Abstracts International
Lafrance, Warman and Woolley (2009) [29]	Sexual identity and the marriage premium	Queen’s Economics Department Working Paper
Lewis (2012) [30]	Remapping disclosure: gay men's segmented journeys of moving out and coming out	Social & Cultural Geography
Lewis and Mills (2016) [31]	Seeking Security: Gay Labour Migration and Uneven Landscapes of Work	Environment and Planning A
Macdonnell and Grigorovich (2012) [32]	Gender, work, and health for trans health providers: a focus on transmen.	ISRN nursing
Mallon (2001) [33]	Oh, Canada: The experience of working-class gay men in Toronto.	Journal of Gay & Lesbian Social Services
Mueller (2014) [34]	Wage differentials of males and females in same-sex and different-sex couples in Canada, 2006–2010	Canadian Studies in Population
Nazaretian (2014) [35]	Social status, opportunity and repeat victimization: The unequal distribution of safety.	Dissertation Abstracts International
Ross et.al. (2018) [36]	In spite of the system: A qualitatively-driven mixed methods analysis of the mental health services experiences of LGBTQ people living in poverty in Ontario, Canada.	PLOS One
Waite and Denier (2015) [37]	Gay pay for straight work: Mechanisms generating disadvantage.	Gender & Society
Waite (2015) [38]	Does it get better? A quasi-cohort analysis of sexual minority wage gaps.	Social Science Research
Waite and Denier (2016) [39]	Self-employment among same-sex and opposite-sex couples in Canada.	Canadian Review of Sociology
Wells (2018) [40]	Transgender Teachers: The Personal, Pedagogical, and Political	Journal of Homosexuality

## Results

There is a considerable body of literature exploring LGBTQ2S+ employment, labour markets, and income in Canada (n = 31 studies). This literature is relatively recent, with 77% (24/31) of the articles published after 2010. This literature can be separated into three broad categories. The first category includes studies that have used population-based surveys to study the employment *outcomes* of the LGBTQ2S+ community, including human capital, labour supply, occupation and industry, and the earnings of sexual minorities, relative to their heterosexual counterparts. The goal of this research is to produce generalizable results about the LGBTQ2S+ community. The biggest limitation for this research has been the availability of population-based surveys that ask questions on sexual orientation, gender identity and employment characteristics [41]. At this time, there are seven population-based surveys that gather data on sexual orientation and three that include questions on non-binary gender in Canada [41]. Table 2 provides an overview of the current LGBT Canadian data landscape. These data limitations have meant that research on the LGBTQ2S+ community has emerged unevenly. To date, most research has focused on coupled gay men and lesbians identified through the census. This approach assumes individuals in same-sex partnerships are either gay or lesbian and, in doing so, erases the possibility of bisexual identifies. We discuss this issue further below.

**Table 2:** Canada’s LGBT data landscape.

Self-reported sexual orientation question	Nonbinary gender option or transgender question	Couple data^a^
Canadian Community Health Survey (CCHS), since 2003	Survey of Sexual Misconduct in the Canadian Armed Forces (SSMCAF), 2016 and 2018	Census of Canada, 2001, 2006, 2016
General Social Survey (GSS), since 2004 but only select years	Public Service Employee Survey (PSES), 2017 and 2018	National Household Survey (NHS), 2011
Canadian Alcohol and Drug Use Monitoring Survey (CADUMAS), since 2008	Survey on Opioid Awareness (SOA), 2017	Longitudinal Administrative Databank (LAD)
Canadian Longitudinal Study of Aging (CLSA), 2010		
Survey of Sexual Misconduct in the Canadian Armed Forces (SSMCAF) 2016 and 2018		
Canadian National Health Survey (CNHS), 2016^b^		
Public Service Employee Survey (PSES), 2018		

See Waite and Denier [41] *A Research Note on Canada’s LGBT Data Landscape* for a thorough review.

^a^ This is not an exhaustive list. It may be possible to identify same-sex couples in other surveys or administrative data sets.

^b^ This was a one-time survey. The data is not currently available for academic use.

The second body of literature has explored the employment *experiences* of the LGBTQ2S+ community, using convenience sampling and semi-structured interviews. This literature provides insight into the subjective employment experiences of LGBTQ2S+ individuals. Unlike the previous category, this literature does not strive for generalizability. Often, these studies isolate a particular occupation or field and unpack how sexuality operates within a particular workplace culture. Examples include gay men and lesbians in the military or policing services [21,27], transgender men in healthcare [32] or teaching [40]. One study [10] used an experimental audit methodology to determine whether law firms have a preference for interviewing heterosexual job applicants over gay and lesbian signaled applicants. Similar to the employment *outcomes* literature, this body of work has also focused predominantly on gay men and lesbians, excluding bisexual, transgender, queer and two-spirit individuals.

The third body of literature is a catch-all category for studies that tangentially capture information on employment outcomes and experiences of the LGBTQ2S+. The majority of these studies are focused on health outcomes between heterosexuals and LGBTQ2S+ individuals and gather employment and earnings information as control variables, rather than a substantive outcome.

### LGBT employment outcomes

It was not until the early 2000s that Canadian surveys began to ask questions on sexual orientation [41]. Carpenter [18] was among the first to use these new data to explore whether gay men and lesbians in Canada had earnings that differed from their heterosexual counterparts. Using the 2003 and 2005 Canadian Community Health Survey (CCHS), he found that gay men had personal incomes that were 12% less than heterosexual men and lesbians had personal incomes that were 15% higher than heterosexual women. Carpenter [18] then compared these findings to estimates obtained using couple data from the 2001 Canadian Census. He found much larger wage gaps when using couple data and argued that the couple approach to estimating sexual minority wage gaps may overstate the magnitude of sexual minority wage gaps. Using similar data, LaFrance, Woolley, and Warman [29] explored the effect of marriage and cohabitation on gay, lesbian, bisexual and heterosexual individuals’ hours worked and full-time earnings. They found that gay men worked fewer hours and earned less than heterosexual men. Lesbians worked longer hours and had higher incomes. Married heterosexual men earned more than everyone else, including common-law and single heterosexual men. Married and common-law gay men did not earn much more than single gay men. One of the most startling findings was the poor labour market outcomes for both bisexual men and women. Bisexual men earned less than both heterosexual men and gay men. Bisexual women were at the bottom of the gender and sexual orientation wage hierarchy. Surprisingly, few subsequent studies have explored the weak labour market outcomes of bisexuals.

Cerf [19] (see Harris [28] for an unpublished version of this article) also pooled cycles of the CCHS to determine whether sexual minority wage gaps could be explained by differences in individuals’ preference for workplace stress. He found that partnered gay men had incomes that were 13% less than heterosexual men but that they also had less stressful workplaces. Partnered lesbians earned 8% more than heterosexual women but they also had more stress at work. Single gay men but not single lesbians, experienced far more stress at work than their heterosexual counterparts. Cerf [19] argued that single gay men’s workplace stress may be associated with anxiety about disclosing their sexuality; whereas, coupled gay men are more likely to be open about their sexual orientation at work. His theoretical model suggests that coupled gay men’s higher household incomes allow them to trade earnings for less stressful workplaces. Lower lesbian household incomes may require that lesbians be willing to take on more stressful jobs with higher earnings to supplement their household incomes.

Dilmaghani [24] pooled repeated cycles of the 2008 to 2012 Canadian Alcohol and Drug Use Monitoring Survey (CADUMS) to estimate sexual minority wage gaps. She found that full-time employed lesbians earned roughly 12% more than heterosexual women. Full-time employed gay men had earnings that were statistically indistinguishable from heterosexual men. Dilmaghani [24] also explored differences in household income and found that coupled gay men had the highest household incomes, followed by heterosexuals, and lastly lesbian households. The finding that gay men’s earnings were statistically indistinguishable from heterosexual men is an outlier from previous studies but may be driven by the use of categorical top coded income [23]. Similarly, the CADUMS does not collect information on important income determinants, such as hours worked or occupation. Table 3 provides an overview of LGB wage gap estimates derived from surveys containing direct questions on sexual orientation. To date, there have been no estimates of transgender, queer, or two-spirit earnings in Canada.

**Table 3:** Sexual minority wage gaps in Canada, self-reported sexual orientation.

Author (year)	Data	Dependent variable	LGBTQ2+ groups†	Sample specifications	Gay	Lesbian	Bisexual men	Bisexual women
Carpenter (2008) [18]	CCHS (2003 & 2005)	Annual income	Gay men and lesbian women Estimates for bisexuals not reported.	Ages 18 to 55	-0.115*** -0.084 n/s (single only) -0.210*** (partnered only)	0.154*** 0.012 n/s (single only) 0.359*** (partnered only)	Less likely to be working full-time and incomes less than heterosexual men (footnotes 17 & 18).	Less likely to be working full-time and incomes less than heterosexual women (footnotes 17 & 18).
Cerf (2016) [19]	CCHS (2003–2009)	Hourly income: annual income divided by 50 x hours worked/week	Gay men, lesbian women Excludes bisexual.	Ages 18–65, Canadian born, Non- Aboriginal, not self-emp. full-time	-0.090 n/s -0.130** (partnered only)	0.027 n/s 0.079* (partnered only)	N/A	N/A
Dilmaghani (2018) [24]	CADUMS (2008–2012)	Annual personal and household income (categorical)	Gay men, lesbian women Excluding bisexuals.	Full-time	0.045 n/s 0.103 n/s (partnered only) 0.299*** (household income, ref = heterosexual households) 0.263*** (likelihood of being in high income hh)	0.116** 0.090 (partnered only) -0.253* (household income, ref = heterosexual households -0.121* (likelihood of being in low income hh)	N/A	N/A
LaFrance, Warman, and Wooley (2009) [29]	CCHS (2003–2007)	Annual income	Gay men, bisexual men, lesbian women, bisexual women	Ages 25 to 59, working 30+/per week	-0.258** (single only) -0.217** (partnered only)	0.048 n/s (single only) 0.100* (partnered only)	-349** (single) -0.281** (married / common-law)	-0.036 n/s (single) -0.141** (married / common-law)

Notes:

*p< 0.05

**p< 0.01

***p< 0.001.

†No data on transgender, queer, two-spirited etc. individuals.

To overcome the problem of small samples and, in some cases, less than ideal employment measures, researchers often turn to couple data, such as the Census, to isolate married and cohabiting gay men and lesbians. Census data contains information on thousands of same-sex couples, high quality earning/income, and employment information. Unfortunately, the biggest drawback when using census data is that there is no information on single gay men or lesbians. The couple approach also engages in methodological bisexual erasure, i.e. bisexuals are considered heterosexual or gay/lesbian depending on their partner’s sex. Similarly, the census data does not gather information on transgender, queer, two-spirit or other gender or sexual minority populations. Table 4 provides an overview of sexual minority wage gap estimates derived from couple data.

**Table 4:** Sexual minority wage gaps in Canada, couple data.

Author (year)	Data	Dependent variable	LGBTQ2+ groups†	Sample specifications	Coupled gay men	Coupled lesbian women
Brown (1998) [16]	Census (1991)	Annual employment income	Non-relatives of the same-sec residing together, i.e. coupled gay men and coupled lesbians	Ages 45 to 64; full-time; full-year	30.2% less than married heterosexual men (aged 45 to 54) 22.3% less than married heterosexual men (aged 55 to 64)	23.1% more than married heterosexual women (aged 45 to 54) 18.9% more than married heterosexual women (aged 55 to 64)
Denier and Waite (2016) [23]	Census (2006)	Annual earnings (continuous & categorical); annual income (continuous & categorical); hourly earnings (continuous & categorical); hourly income (continuous and categorical)	Married / cohabiting gay men and lesbians	The authors replicate sample specifications in Mueller (2014) and Waite and Denier (2015). This paper attempts to reconcile the differences in point estimates from the GSS and Census data	Between -0.126*** and -0.051*** depending on whether income or earnings; continuous vs. categorical; with or without controls	Between 0.095*** and 0.035** depending on whether income or earnings; continuous vs. categorical; with or without controls
Denier and Waite (2017) [22]	Census (2006)	Annual wages and salaries	Married / cohabiting gay men and lesbians	Ages 25 to 64, annual earnings $1000+	-0.077*** (National) -0.083*** (Montreal) -0.064** (Toronto) -0.080** (Vancouver) -0.090*** (Other cities) -0.177*** (non-city)	Note: ref = coupled heterosexual men -0.130*** (National) -0.097*** (Montreal) -0.107*** (Toronto) -0.173*** (Vancouver) -0.142*** (Other cities) -0.159*** (non-city)
Mueller (2014) [34]	GSS (2006–2010) ^+^	Hourly income: mid-point values of each category for personal income divided by annual hrs worked	Married / cohabiting gay men and lesbians	Ages 20–60, not attending school full-time, between $5 and $500/hr and claimed employment or self employment income if main source of income	-0.060 n/s	0.163***
Waite and Denier (2015) [37]	Census (2006)	Annual wages and salaries	Married / cohabiting gay men and lesbians	Ages 25–64, Canadian born, non-Aboriginal, non-visible minority, annual earnings $1000+, working for wages and salaries	-0.051*** (see article appendix) -0.74*** (private sector employment) 0.007 n/s (public sector employment)	0.079*** (see article appendix) 0.091*** (private sector employment) 0.050*** (public sector employment)
Waite and Denier (2016) [39]	Census (2001, 2006) & National Household Survey (2011)	Annual wages and salaries	Married / cohabiting gay men and lesbians	Ages 25 to 64, Canadian born, non-aboriginal, excluding self-employed farmers, annual wages and salaries	-0.066*** Wage gaps vary by occupation and are largest in sales and services (-0.136***) and manufacturing, trade, and primary processing (-0.130***).	0.066*** (vs. hetero women) -0.133*** (vs. hetero men) Wage gaps (vs hetero women) vary by occupation and are largest in sales and services (0.175***) and manufacturing, trade, and primary processing (0.208***). Wage gaps (vs hetero men) also vary by occupation and are largest in management (-0.196***), sales and service (-0.176***), and manufacturing, trade, and primary processing (-0.156***).
Waite (2015) [38]	Census (2001, 2006) & National Household Survey (2011)	Weekly wages and salaries: annual wages and salaries / total weeks worked	Married / cohabiting gay men and lesbians	Ages 25–64, Canadian born, non-Aboriginal, non-visible minority, annual earnings $1000+, working for wages and salaries	-0.057*** (in 2001) -0.061*** (in 2006) -0.057*** (in 2011) Those with partners that don’t work or worked part-time: -0.078** (in 2001) -0.077** (in 2006) -0.109*** (in 2011)	0.052*** (in 2001) 0.087*** (in 2006) 0.058*** (in 2011) Those with partners that don’t work or worked part-time: 0.077** (in 2001) 0.080** (in 2006) 0.056* (in 2011)

Notes:

*p< 0.05

**p< 0.01

***p< 0.001.

†No data on bisexual, transgender, queer, two-spirited etc. individuals.

^+^ The confidential GSS data includes a direct question on sexual orientation.

Mueller [34] used public use GSS data, which only includes information on coupled gay men and lesbians

Brown [16] was the first to use Canadian Census data to explore sexual minority wage gaps. Using the 1991 Canadian Census, Brown [16] classified individuals as possibly gay or lesbian when they had a same-sex, non-relative adult living in their household. Although this method for identifying gay men and lesbians was subject to considerable bias, it was the only method available at the time because Statistics Canada did not start gathering information on same-sex couples until 2001. She found that gay men earned less than heterosexual men and lesbians earned more than heterosexual women. The size of these wage gaps varied by age group and whether comparisons were made to married, separated, divorced or widowed heterosexuals.

Since 2001, Statistics Canada has been collecting information on same-sex couples. This overcomes the bias in the methodology employed by Brown [16]. Using the 2006 Canadian Census, Waite and Denier [37] found interesting socio-demographic, human capital and occupational differences between coupled gay men and lesbians, relative to their heterosexual counterparts. For example, they found that gay men and lesbians were slightly younger, more highly educated, had fewer years of potential experience, less likely to be married and/or have children, and more likely to reside in large cities (Toronto, Vancouver and Montreal). They were also more likely to work in gender atypical occupations and industries. For example, gay men were less likely to be working in primary processes, construction, and manufacturing. Lesbians were more likely to work in traditionally male occupations. After controlling for these differences in a multivariate regression model, gay men earned roughly 5% less and lesbians earned 8% more than their heterosexual counterparts. When comparing lesbians to heterosexual men, they earned roughly 9% less. They also found that wage gaps were significantly reduced in Canada’s public sector for gay men, lesbians and heterosexual women. In a subsequent study, Waite and Denier [39] then added the 2001 Canadian Census and the 2011 National Household Survey (NHS), to their sample and explored whether there were differences in the propensity for self-employment by sexual orientation. In particular, they were interested in whether barriers in paid employment increased the attractiveness of being self-employed for sexual minorities. They found that gay men were less likely and lesbians more likely to be self-employed, relative to their heterosexual counterparts. The authors also explored whether sexual minority wage gaps and the propensity for self-employment varied by occupation. They found that gay men and lesbians had the largest wage gaps, relative to their heterosexual counterparts, in sales and service and manufacturing, trades and primary industries. Gay men were more likely to be self-employed in arts and culture and sales and service but less likely to be self-employed in business and finance. Lesbians were more likely to be self-employed in health related occupations, natural and science, and arts and culture occupations.

Waite [38] extended the previous analysis by using data from the 2001 and 2006 Canadian Censuses, as well as data from the 2011 NHS to explore whether sexual minority wage gaps narrowed over the first decade of the twenty-first century. He found little change in sexual minority wage gaps over this period. In addition, wage gaps appeared to be largest for young gay men and smallest for older gay men. The lesbian wage advantage was absent in younger ages and only appeared for older cohorts, suggesting that the lesbians’ higher earnings relative to heterosexual women only appears after many years in the labour market. One explanation may be that heterosexual women’s higher rates of marriage and childbearing have negative effects on their earnings, relative to lesbian women.

Sexual minorities are concentrated in large cities, especially Toronto, Vancouver and Montreal [22]. Denier and Waite [22] were interested in whether estimates of sexual minority wage gaps varied across these areas. In particular, they investigated whether wage gaps in Montreal, Toronto and Vancouver were smaller than those in rural Canada. Using data from the 2006 Canadian Census, they found that gay men’s wage gap was largest in rural Canada (18%) and smallest in Toronto (6%). For lesbians, the largest wage gaps relative to heterosexual men was found in Vancouver (17%) and the smallest was in Montreal (10%).

Mueller [34] pooled public-use data from the 2006 to 2010 General Social Survey (GSS) and found that coupled gay men had earnings that were statistically indistinguishable from heterosexual men. Coupled lesbians earned roughly 18% more than heterosexual women. Some caution needs to be exercised with interpreting these result due to small samples sizes (90 gay men, 118 lesbians). Using similar data, Dilmaghani [25] explored differences in labour supply by sexual orientation. She found that lesbians had a greater labour supply and gay men a weaker labour supply, than their heterosexual counterparts. Dilmaghani [25] also found that gay men and lesbians sorted into gender atypical occupations. Lastly, Allen [12] used couple data from the 2006 census to explore household production by sexual orientation. Of particular note, he found heterosexual households have roughly the same production value, regardless if there are children or no children in the household (roughly $48,000). For gay and lesbian household, the presence of children made a considerable difference in the estimated value of household production. The average value of household production for gay couples with children was $79,256 and $24,712 for those without children. For lesbians with children, the average value of household production was $36,997 and $53,033 for those without children.

The studies outlined above have found considerable variation in the size of sexual minority wage gaps. For example, Brown’s [16] descriptive analysis found that gay men earned between 6% and 30% less than heterosexual men; whereas, lesbians earned between 6% and 26% more heterosexual women. These estimates varied based on the heterosexual comparison group, i.e. when comparing opposite-sex marriage men to same-sex men the wage ratio was 130.2% for heterosexual men between the ages of 45 to 54. Carpenter [18] used the 2003 and 2005 CCHS and found that gay men earned 12% less than heterosexual men and lesbians earn 15% more than heterosexual women. Using the 2006 Canadian Census, Waite and Denier [37] found that coupled gay men earned 5% less and lesbians earned 8% more than their heterosexual counterparts. Mueller [34] found that lesbians earned 18% more than heterosexual women in the GSS, while Dilmaghani [24] found that lesbians earned 12% more using the CADUMS. Both studies did not find a statistically significant wage gap for gay men. In an attempt to reconcile these inconsistencies, especially the null finding for gay men in the Mueller [34] study, Denier and Waite [23] explored how estimates of sexual minority wage gaps could be sensitive to the types of income / earnings measures and sample specifications used; for example, using total wages and salaries vs. total personal income, categorical income vs. continuous and top-coded vs. non-top-coded income. While they found considerable heterogeneity in sexual minority wage gap estimates depending on how wages and income were measured, the general pattern observed was that coupled gay men earned less and coupled lesbians earned more than their heterosexual counterparts.

Canada’s General Social Survey (GSS) began asking a question on sexual orientation in 2004. To date, only one study using the GSS provides us any information in sexual minority employment and earnings. Using the 2004 and 2009 victimization cycles of the GSS, Nazaretian [35] found that lesbian, gay, and bisexual (LGB) individuals had higher rates of victimization, relative to heterosexuals, Aboriginals, and visible minorities. Due to small sample sizes, the author was unable to disaggregate the LGB group. Relevant to this review, the authors also provide some descriptive analysis of income. Nazaretian [35] found that aboriginal and visibility minorities had the lowest incomes, followed by LGB individuals and women. Men reported the highest incomes.

The literature on transgender employment outcomes in Canada is considerably underdeveloped. This is primarily due to the dearth of population-level data that include questions on transgender and other non-binary identities. Currently, there are three Canadian population-level surveys that capture information on non-binary gender identities and relevant employment characteristics [41]. Data from the 2017 Public Sector Employee Survey (PSES), found that 35% of gender diverse federal employee experienced harassment in the workplace, compared to 16% and 19% of cisgender men and women. Gender diverse respondents were those that answered “other” to the gender question (i.e. male; female; other, please specify). Gender diverse employees, which includes transgender, genderqueer and gender non-binary individuals, also report lower levels of workplace satisfaction, feel that they receive less meaningful recognition, are less valued, and less respected in the workplace, relative to cisgender male and female employees [42]. The Survey of Sexual Misconduct in the Canadian Armed Service (SSMCAF) includes a self-identity sexual orientation question, as well as a gender identity question. Using the 2016 SSMCAF, Cotter [20] found that LGBT Regular Force members were more likely to report workplace discrimination and sexual assault, relative to non-LGBT Regular Force Members [20]. The third survey that collects information on non-binary gender identities is the Survey or Opioid Awareness (SOA) but this contains no valuable employment information. To date, there are no population-level surveys that allow researchers to produce meaningful estimates of transgender or other non-binary individual’s wages.

In the most comprehensive study of transgender Canadians to date, Bauer and Scheim [13] gathered information on sociodemographic characteristics, employment discrimination, educational barriers, health care access, violence, and mental and physical health of 433 transgender individuals living in Ontario. Their study also included focus groups with transgender community members and family members from their sample. Of particular interest to our systematic review, they found that 13% of transgender Ontarians reported that they were fired for being transgender and another 15% were fired for reasons they believed were related to their gender identity. While this was not a population-level survey, the authors did take steps to address generalizability. For example, they used respondent-driven sampling (RDS), which is a methodology used to improve generalizability when gathering a random sample is not feasible.

While these studies have identified important differences in human capital, occupation, earnings, and even self-reported employment discrimination and harassment by sexual orientation or gender identity, another branch of literature has been interested in studying subjective *experiences*. This literature has relied on semi-structured interviews and convenience samples of LGBTQ2S+ individuals. More than half of the articles with keywords for employment, labour markets and earnings are studies about the employment experiences of LGBTQ2S+ individuals.

### LGBTQ2S+ employment experiences

The second body of literature focuses on LGBTQ2S+ employment *experiences*. Unlike the previous literature, the focus here is on the subjective experiences of LGBTQ2S+ peoples in the Canadian labour market. This literature has been interested in unpacking how workplace cultures or local contexts aid or hinder the inclusion of minorities in the workplace. This also includes research on employment discrimination against LGBTQ2S+ community.

The first Canadian study to explore workplace discrimination against gay men and lesbians came two decades before population-based surveys started asking questions about sexual orientation. Adam [10] conducted the first audit study of employment discrimination against gay male and lesbian job seekers. In his study, identical resumes were sent to Ontario law firms signaling gender by using male and female sounding first names and sexual orientation by including volunteer experience on a “Gay People’s Alliance”. He found that gay and lesbian signaled job applicants received fewer interview offers than non-signaled, ostensibly heterosexual, job candidates. Non-labelled male applicants received 1.6 times as many interview offers than the gay-signaled applicants. Non-labelled female job seekers received twice as many interview offers than lesbian-signaled job applicants. This study provided the first evidence of employment discrimination against gay- and lesbian-signaled job seekers in Canada.

Most other studies have used semi-structured interviews to gather information on the subjective workplace experiences of the LGBTQ2S+ community. Bowring and Brewis [14] interviewed 16 gay and lesbian workers in three Canadian cities to better understand how they managed their non-hegemonic identities at work. The authors found that organizational environments are important for helping sexual minorities integrate their identities into the workplace. Being in a same-sex relationship also made the coming out process at work easier. Other studies have focused on LGBTQ2S+ employment experiences within a particular occupation or field, which the author(s) argue provide an interesting site for analysis [21,27,32,33,40]. For example, MacDonnell and Grigorovich [32] conducted semi-structured interviews with four Canadian transmen working in the healthcare profession to better understand the links between work, career, and health. Transmen in healthcare felt that their trans identity was invisible in education programs and the workplace. “Lack of fit” was used by employers to justify discrimination and thwart promotions. Wells [40] interviewed three male-to-female transgender teachers who transitioned while teaching in three different decades (1980s, 1990s, and 2000s). Using post-structural storylines, Wells [40] demonstrates how each participant challenges traditional discourses of transgender invisibility, silence, shame and fear.

In another study, Fournier [27] conducted interviews with 21 gay and lesbian individuals working in the heavily male dominated military and police services in Quebec. She found that while military and police institutions had made considerable progress in the acceptance of homosexuality in recent years, there remains an organizational culture characterised by male chauvinism that can restrict gay and lesbian’s full integration, especially in the military. Gay and lesbian police officers described their experiences as positive with some concerns about displays of homosexuality in the work environment. Couto [21] interviewed 21 LGBT police officers working in Ontario and also found that officers believed that workplace conditions had greatly improved over the past few decades. However, similar to the findings in Fournier [27], respondents still felt that the police service retained a hypermasculine, conservative, male-dominated, and heteronormative culture, where the archetypal cop was at times inconsistent with their personal sexual lives.

Mallon [33] interviewed 10 working-class men living in Toronto to better understand how social class shaped their identity as gay men. The author argues that gay social discourse has focused predominantly on middle class gay men, excluding working class blue-collar gay men. The study found that appearance, participation in the Toronto Bear culture and the role or work were instrumental in their identity as gay men.

Three studies have taken steps to incorporate sexuality into the migration literature by exploring the motivations and experiences of migration for sexual minorities [11,30,31]. Lewis and Mills [31] conducted a cross-national study of work-related migrations of gay-identified men (n = 48) living in Ottawa, Ontario and Washington, DC. The goal of this study was to better understand how sexuality impacted work-related migration decisions of gay men. Amongst other things, their study highlighted the importance of sexuality in the migration decisions of gay men. For many, the decision to move was impacted by intolerance and gay-related stigma in their current city and/or job. Moving fulfilled multiple needs for gay men, including escaping homophobic environments, finding employment in more tolerant workplaces, and the social benefits of moving to a city with a larger gay population. In an earlier paper, Lewis [30] used similar data to challenge the linearity of the coming-out migration literature, arguing that the journey from migration to coming-out was far more complex than previously theorized. While his paper does not focus on employment experiences, it does demonstrate how migrants must negotiate the coming out process across various landscapes, including the workplace. Adam and Rangel [11] studied the post-migration experiences of 25 Spanish speaking gay and bisexual men living in Toronto. Participants were asked questions about their pre- and post- migration experiences in the workplace and gay communities, push and pull factors for migration, their sense of inclusion/exclusion, and other barriers experienced in Canada. Sexuality played an important role in migration experiences. For many respondents, homophobia was a push factor and Canada’s legal protections and marriage equality was a pull factor. Many of the migrants in the study were well educated and had considerable employment experience but faced barriers transitioning into the Canadian labour market.

### Tangential employment or earnings data

The third body of literature is a catch-all category for studies tangentially gathering information on LGBTQ2S+ human capital, employment, occupation, or earnings. These studies may use surveys but are typically limited in their generalizability because they focus on specialized sub-populations. This research also tends to use data gathered on subpopulations who access physical, mental, or sexual health-related services. For example, Card et.al. [17] used a respondent-driven survey of alcohol and drug use by gay, bisexual, and/or transgender men to identify six classes of men who consume alcohol and other drugs. The survey also gathered data on employment and earnings. The authors found that men in the ‘street drug use’ category were more likely to be out of work and less likely to be stably housed than those in the “club drug use’ or ‘conventional drug use’ groups. In another health survey, Farlatte et.al. [26] explored the syphilis epidemic affecting Canadian gay and bisexual men who have sex with other men (GBMSM). Amongst other predictors, they found that anti-gay stigma, which included career discrimination in the last 12 months, was a significant predictor of syphilis. The authors suggest that stigma and minority stress may play a role in GBMSM syphilis transmission. Brennan et.al. [15] used data from the Ontario HIV Treatment Network Cohort Study to study the sociodemographic characteristics of older people (>50 years) with AIDS/HIV. The survey collected information on education, income, employment and occupation and reports separate results for women, heterosexual men, gay men and bisexual men. Due to small samples, the authors pooled lesbian, gay and bisexual women into a single “women” category. The descriptive results found that older gay men and bisexual men living with HIV/AIDS were more highly educated and had higher incomes than older heterosexual men living with HIV/AIDS. Gay men were the most likely to be employed amongst this older sample; whereas, heterosexual men were the least likely to be employed. Gay men, and bisexual men to a lesser degree, were more likely to be working in senior or middle management, or professional occupations than heterosexual men. Older gay men and women had better self-reported health, compared to bisexual and heterosexual men. Given their education and higher incomes, the authors conclude that older gay and bisexual men living with HIV/AIDs were doing fairly well economically, compared to their heterosexual counterparts.

Ross et.al. [36] conducted a qualitatively-driven mixed methods analysis of mental health service experiences of LGBTQ people living in Ontario. A subsample of LGBTQ individuals who completed an online survey related to experiences of depression were questioned using semi-structured interviews. They found that low income LGBTQ respondents had more visits with mental health professionals and more unmet mental health needs than either higher income LGBTQ respondents or low income heterosexual, cisgender respondents. Their qualitative results found higher levels of mental health care utilization for LGBTQ people living in Ontario, relative to heterosexuals, but either equivalent or lower levels for low income LGBTQ individuals. The authors suggest that the inaccessibility of private mental health care services for low income LGBTQ individuals may explain this finding.

## Discussion and conclusion

The biggest limitation for researchers interested in studying the labour market outcomes of the LGBTQ2S+ communities continues to be the dearth of high-quality data that includes questions on sexual orientation, non-binary gender identity and relevant employment variables. The LGBT population is relatively small [43–46], which requires large population-based surveys to produce meaningful samples for multivariate analysis. Unfortunately, there are no high-quality estimates of the prevalence of queer, two-spirit, or other (Q2S+) individuals in the population. The vast majority of literature on LGBTQ2S+ employment outcomes has focused on coupled gay men and lesbians. Data that include direct questions on sexual orientation allow researchers to identify single gay men and lesbians, as well as bisexual individuals but sample sizes are often small or contain few relevant employment measures. These limitations make it particularly difficult to explore the intersections of gender, sexual orientation, race and class. To date, no study has systematically explored these issues. The recent findings that bisexual men and women are particularly disadvantaged in the labour market warrants further attention. In particular, more research is needed to better understand the mechanisms driving bisexual men and women’s weak labour force participation and lower earnings.

While the literature on gay, lesbians and even bisexual employment outcomes has grown in recent years, there has been almost no literature on transgender, queer, two-spirit and other gender and sexual minority groups. Again, data limitations hinder research in this field. Perhaps the availability of new surveys with questions on gender identity will provide opportunities for researchers. For example, Statistics Canada has recently signaled that the 2021 census will include a non-binary gender response option. As more surveys move in this direction, new research avenues will emerge.

With the exception of Waite and Denier [22], there has been little research exploring geographic variations in employment experiences and outcomes. Regional differences may be particularly important if workplace cultures and social attitudes emerge at the local level. For example, LGBTQ2S+ employment experiences and outcomes in predominantly white-collar Toronto might be different from in rural Alberta where oil and gas industries are dominate.

While research on sexual minority wage gaps and employment outcomes is constrained by the availability of data, research on the employment experiences of LGBTQ2S+ peoples is not. Qualitative research provides valuable insights into the lived experiences of LGBTQ2S+ individuals. Surprisingly, there has been relatively little research exploring these issues in Canada. Few researchers have extended their analyses on LGBTQ2S+ identity to work and the labour market. At the same time, the research that does exist also seems to focus on particular segments of the LGBTQ2S+ community, i.e. gay men, lesbians and transgender individuals. There are few qualitative studies on bisexuals and other gender and sexual minorities.

This review of the LGBTQ2S+ literature has also highlighted a complete absence of research on two-spirit individuals’ labour market outcomes and experiences. The intersection of Indigeneity, gender identity and sexual orientation may be particularly important. We know that Canada’s Indigenous people face considerable barriers in the labour market [47] but we know very little about the employment experiences of two-spirit peoples. Given the current data landscape and marginalization of the Indigenous population, researchers will likely need to rely on inductive methods to answer these questions.

Our systematic review has highlighted important differences in employment outcomes and experiences for Canada’s LGBTQ2S+ communities. The majority of this literature falls into two broad categories. The first category includes studies drawing from population-based surveys to study the employment *outcomes* of the LGBTQ2S+ community, with the goal to produce generalizable results. The second focuses on LGBTQ2S+ employment *experiences*. This literature explores subjective experiences, rather than generalizable outcomes. It also unpacks how workplace cultures or local contexts aid or hinder the inclusion of minorities in the workplace.

Starting with the literature on employment outcomes, we find important differences in employment and earnings by sexual orientation. With few exceptions, studies find that gay men earn less and lesbians earn more than their heterosexual counterparts. Bisexual men and women appear to fare the worst in this gender and sexual orientation hierarchy. This literature has also found important differences in wage gaps across cities and by partnership type. Estimates of sexual minority wage gaps derived from couple data may be larger than estimates from surveys that include direct measures of sexual orientation. Sexual minority wage gaps are larger in non-urban areas than in Toronto, Montreal and Vancouver. Gay men and lesbians appear to sort into gender-atypical occupations and industries. There may also be differences in the type of employment sexual minorities prefer, such as self-employment.

The second branch of literature, which focuses on subjective employment experiences, finds important employment barriers for LGBTQ2S+ people. This is especially the case for racialized minorities and immigrants. Surprisingly, this literature is less developed than research on sexual minority employment outcomes. This is surprising given that the latter is dependent on population-based survey data. Both of these literatures have focused on very predominantly on gay men and lesbians, with some limited research attention to bisexual and transgender individuals. There is an absence of research on queer, two-spirited and other sexual minority populations.

This review highlights the ongoing barriers for gender and sexual minorities in Canada. Unlike other countries, Canada has a strong antidiscrimination framework that protects individuals from employment discrimination on the basis of sexual orientation and gender identify. The continued marginalization LGBTQ2S+ individuals suggests that greater efforts need to be taken. One strategy may be to include sexual orientation and gender identity in the Federal Employment Equity Act. This act goes beyond standard antidiscrimination legislation and requires that federal employers take steps to foster inclusion of minority employees in the workplace.

## Supporting information

S1 Checklist (DOC)

S1 Appendix (DOCX)
