# Supplementary material for: A systematic review and thematic synthesis of Canada’s LGBTQ2S+ employment, labour market and earnings literature
Source: PLoS One. 2019 Oct 2;14(10):e0223372. doi: 10.1371/journal.pone.0223372 (PMC6774499; doi:10.1371/journal.pone.0223372)
Supplement: S1 Appendix — (DOCX) [file pone.0223372.s002.docx]

| **S1 Appendix. Systematic Review Database Search Keywords** | | |
| --- | --- | --- |
| ***LGBTQ2S+-related terms*** | ***Poverty-related terms*** | ***Canada-related terms*** |
| “Sexual orientation” (SU)  “Sexual minority” (SU)  “Sexual identity” (SU)  “Sex role identity” (SU)  Bisexual* (SU)  Lesbian* (SU)  Homosexual* (SU)  “Sexual preference*” (SU)  “Sexual behaviour” OR “sexual behavior” (SU)  “Gays & lesbians” (SU)  “Transgender persons” (SU)  “Gender identity” (SU)  Transsexual* (SU)  “Sexual minority” (title/abstract)  Lesbian* (title/abstract)  Gay (title/abstract)  Bisexual* (title/abstract)  Non-monosexual OR nonmonosexual (title/abstract)  Plurisexual* (title/abstract)  Men who have sex with men  (title/abstract)  MSM* (title/abstract)  Women who have sex with women (title/abstract)  WSW* (title/abstract)  Transgender* (title/abstract)  Transsexual* (title/abstract)  Queer (title/abstract)  Two-Spirit* (title/abstract)  LGB* (title/abstract)  GLB* (title/abstract)  “Sexual orientation” (title/abstract)  “Gender identity” (title/abstract) | Poverty (SU)  “Homeless people” (SU)  Homelessness (SU)  “Low income groups” or “low income” (SU)  Poor (SU)  “Wage gap*” (SU)  Education* (SU)  Employ* (SU)  Unemploy* (SU)  Welfare (SU)  Shelters (SU)  Income (SU)  “Financial support” (SU)  Wealth (SU)  Housing (SU)  “Antipoverty programs” (SU)  Deprivation (SU)  Social class (SU)  “Socioeconomic status” (SU)  Occupation* (SU)  Poverty (title/abstract)  Poor (title/abstract)  “Wage gap*” (title/abstract)  Income (title/abstract)  Unecmploy* (title/abstract)  Employ* (title/abstract)  Homeless* (title/abstract) | Canad* (SU)  Toronto (SU)  Montreal (SU)  Ontario (SU)  Quebec (SU)  Vancouver (SU)  “British Columbia” (SU)  Alberta (SU)  Manitoba (SU)  Saskatchewan (SU)  “New Brunswick” (SU)  “Nova Scotia” (SU)  “Prince Edward Island” (SU)  Newfoundland* (SU)  Yukon (SU)  “Northwest Territories” (SU)  Nunavut (SU)  Canad* (title/abstract)  Toronto (title/abstract)  Vancouver (title/abstract)  Montreal (title/abstract) |

SU = subject
